# Antimicrobial Doses in Continuous Renal Replacement Therapy: A Comparison of Dosing Strategies

**DOI:** 10.1155/2016/3235765

**Published:** 2016-06-28

**Authors:** Anna P. Kempke, Abbie S. Leino, Farzad Daneshvar, John Andrew Lee, Bruce A. Mueller

**Affiliations:** Clinical Pharmacy Department, University of Michigan College of Pharmacy, Ann Arbor, MI 48109, USA

## Abstract

*Purpose*. Drug dose recommendations are not well defined in patients undergoing continuous renal replacement therapy (CRRT) due to limited published data. Several guidelines and pharmacokinetic equations have been proposed as tools for CRRT drug dosing. Dose recommendations derived from these methods have yet to be compared or prospectively evaluated.* Methods*. A literature search of PubMed, Micromedex, and Embase was conducted for 40 drugs commonly used in the ICU to gather pharmacokinetic data acquired from patients with acute and chronic kidney disease as well as healthy volunteers. These data and that obtained from drug package inserts were gathered for use in three published CRRT drug dosing equations. Doses calculated for a model patient using each method were compared to doses suggested in a commonly used dosing text.* Results*. Full pharmacokinetic data was available for 18, 31, and 40 agents using acute kidney injury, end stage renal disease, and normal patient data, respectively. On average, calculated doses differed by 30% or more from the doses recommended by the renal dosing text for >50% of the medications.* Conclusion*. Wide variability in dose recommendations for patients undergoing CRRT exists when these equations are used. Alternate, validated dosing methods need to be developed for this at-risk patient population.

## 1. Introduction

The use of continuous renal replacement therapy (CRRT) is increasing in the management of acute kidney injury (AKI) [1]. CRRT establishes hemodynamic stability and provides excellent volume and metabolic control in critically ill patients with AKI. Like native kidneys, CRRT also removes drugs in the process of correcting fluid overload and azotemia. However, CRRT does not provide tubular secretion and reabsorption seen in a properly functioning kidney. Further, critically ill patients receiving CRRT often exhibit altered pharmacokinetic parameters that must be accounted for when dosing medications [2, 3]. Appropriate pharmacotherapy is further complicated by the many CRRT methodologies and flow rate settings used clinically.

CRRT drug dosing guidance has always been lacking for clinicians. Although early and appropriate antibiotic therapy has been linked with better patient outcomes in critically ill patients with sepsis [7], the Food and Drug Administration (FDA) does not mandate antibiotic pharmacokinetic trials in CRRT, and rarely do dosing guidelines exist in approved package inserts [8].

Due to the paucity of dosing guidance, many CRRT dosing methods have been proposed and range from guidelines to dosing equations [3, 5, 6, 9–13]. A published reference by Aronoff et al. provides a dosing guideline for patients with renal failure and includes recommendations for CRRT based on available published pharmacokinetic studies and clinical judgment [10]. The antibiotic CRRT doses in this book were developed by one of the authors (BAM) and can be viewed online at https://kdpnet.kdp.louisville.edu/drugbook/adult/. Similarly, equations utilizing drug-specific pharmacokinetic variables have been developed for drug dosing determination (see Table 1) [4–6]. Many of these equation-based methods published in the 1990s are still used by clinicians today as they allow for the calculation of doses using known CRRT parameters and published pharmacokinetic data for nearly any drug, including those never studied in the setting of CRRT. Relatively robust assumptions must be made when using these equations as it is not often clear whether the applied pharmacokinetic data are derived from critically ill patients with their often abnormal pharmacokinetic profiles or if the data applied are from patients with acute or chronic kidney failure. Depending on what parameters are chosen, different doses may be calculated, but this has not been investigated.

A prospective comparison of the published CRRT drug dosing strategies has yet to be done. It is unknown whether the guidelines and equations proposed by various authors over the years result in similar CRRT doses. The purpose of this investigation was to compare the calculated antimicrobial dose recommendations among the different methods and compare them to a published guideline coauthored by one of the authors (BAM). This comparison will not determine which dosing method results in therapeutic serum concentrations; however, it will provide better understanding of the current tools available for drug dosing in CRRT and provide direction for future research. Additionally, the influence of pharmacokinetic assumptions made (i.e., utilization of AKI, stage 5 of chronic kidney disease (CKD5), or normal patient pharmacokinetics) in calculating doses will be assessed.

## 2. Methods

### 2.1. Study Design

This study was a prospective application of currently available pharmacokinetic data to existing dosing methods in order to determine their level of agreement. Ultimately, CRRT doses derived from different formulas and the Aronoff et al. dosing guideline were compared [4–6, 10, 14]. Published works by Reetze-Bonorden et al., Bugge, and Kroh provided the dosing equations (see Table 1). Maintenance doses were calculated for each equation using published pharmacokinetic data from patients with AKI and CKD5 and using data from healthy volunteers in order to determine the influence of using data from different patient populations. Doses calculated with pharmacokinetic parameters collected from AKI, CKD5, or normal renal function patients are denoted as D_AKI_, D_CKD5_, and D_N_, respectively.

### 2.2. Drug Selection

Selection of the antimicrobial agents studied in this trial was based on a paper describing the top 100 drugs used in the ICU at the University of Michigan Hospitals [15]. Additionally, drugs with CRRT doses supported by “human trials larger than a case study” (the highest evidence grade) in the Aronoff et al. text were also included [10].

### 2.3. Pharmacokinetic Variables

The necessary medication variables for each equation were obtained through an extensive literature search. Fraction of drug unbound was used for sieving coefficient as it is an accepted surrogate [16]. Effluent rate was assumed to be 2 liters per hour (33.3 mL/min)—the rate used by Aronoff et al. and a rate consistent with clinical practice. Recommended normal doses were obtained from the prescribing information assuming a patient weight of 70 kilograms. Anuric dose recommendations were also obtained from the prescribing information; however, if the drug was not recommended for use in an anuric patient, no comparisons were made. The Reetze-Bonorden et al. equation was the only one affected by the availability of anuric dose recommendation because it is a required element in the equation [6].

### 2.4. Search Strategy

An extensive literature search included the following: the prescribing information, PubMed, Micromedex, and Embase. Search terms contained the following: end stage renal disease, acute kidney injury, acute renal failure, drug name, pharmacokinetics, the name of the pharmacokinetic variable, CRRT type, and sieving coefficient or apparent sieving factor or saturation coefficient. References cited by papers found through this search were further explored to identify the primary literature.

### 2.5. Data Prioritization

Sieving coefficient, apparent sieving factor, saturation coefficient, or other variations of this constant were all accepted under the general variable “sieving coefficient” due to scarcity of available data. Sieving coefficient may vary depending on CRRT methodology; consequently, CVVH data was given first priority, followed by CVVHDF, and finally CVVHD. When possible, CRRT-related data was derived from studies published in 1990 or later to account for advancing technology (changes in filter types, machines, and settings). Date of publication did not play a role in the use of such references for properties inherent to the drug.

The assumed treatment indication for each studied antimicrobial was the one most likely to be present in the ICU setting as determined by our research team. Generally, “severe” infections were usually assumed and doses to be used in the equations were those used for severe infections. When doses were given as a range, a value that fell approximately at the middle of the range and correlated with the available dosage forms was used. If multiple sources provided discrepant values for a particular pharmacokinetic variable, all data were recorded and the average was taken to determine a single value to be used for the purpose of the dose calculations.* In vivo* data took priority over* in vitro* or animal data, and multiple-dose pharmacokinetic studies took priority over single-dose kinetics. For data collected for CKD5 or AKI pharmacokinetic parameters, the authors must have clearly documented that the patients were anuric. Additionally, all nonrenal clearance values had to be collected when the patient was not undergoing dialysis.

### 2.6. Data Analysis

Doses predicted by published equations were compared to those recommended in the Aronoff et al. dosing guidelines. Aronoff et al. text was the chosen comparator as it is among the most commonly used dosing texts and was coauthored by one of the authors of this study (BAM). Like the other dosing equations, Aronoff et al. equation has not been tested prospectively. Authors of this text applied available pharmacokinetic and pharmacodynamic data at time of publication and used a consensus approach of a group of experts. The percent difference between the Aronoff et al. dose and the calculated dose for each antimicrobial was determined, with an absolute difference of ≥30% considered “clinically significant.”

### 2.7. Statistical Analyses

Each dose was converted into mg/day to allow direct comparisons between methodologies. For each dosing equation, the number of drug doses within ±30% of the Aronoff et al. dose and outside of this range was assessed using the Chi squared test. Chi squared tests also were used to determine whether the assumed pharmacokinetic parameters (AKI versus CKD5 versus normal) affected the number of drug doses within ±30% of the Aronoff et al. dose. Descriptive statistics were used to evaluate the dose differences between the equations and the Aronoff doses.

## 3. Results

Forty antimicrobials were included in this assessment. Full AKI pharmacokinetic data was available for 18 drugs, CKD5 pharmacokinetic data was available for 31 drugs, and normal pharmacokinetic data was available for all 40 agents (see Appendix, in Supplementary Material available online at http://dx.doi.org/10.1155/2016/3235765). Doses were calculated and the agreement or disagreement with the doses recommended by Aronoff et al. was determined (see Table 2).

Of the agents available for dose calculations utilizing the Bugge equation, 55% of D_CKD5_ (dose difference ranged from −100% to 85%, median −22.4%), 59% of  D_AKI_ (dose difference ranged from −100% to 65%, median −30.9%), and 48% of D_N_ (dose difference ranged from −73% to 89%, median −21.4%) fell outside of ±30% of the doses recommended by Aronoff et al. Of the agents available for dose calculations utilizing the Kroh equation, 61% of D_CKD5_ (dose difference ranged from −94% to 68%, median −33.7%), 82% of D_AKI_ (dose difference ranged from −85% to 99%, median −36.5%), and 45% of D_N_ (dose difference ranged from −94% to 88%, median −21.3%) fell outside of ±30% of the dose recommended by Aronoff et al. Of the agents available for dose calculations utilizing the Reetze-Bonorden equation, 63% of D_CKD5_ (dose difference ranged from −69% to 207%, median 16.4%) and 50% of D_AKI_ (dose difference ranged from −88% to 122%, median 0.9%) fell outside of ±30% of the dose recommended by Aronoff et al.

A Chi squared analysis was also performed to determine if the doses being in range (±30% of Aronoff et al. dose) or out of range were affected by whether CKD5, AKI, or normal data was used in the equations. For Bugge and Reetze-Bonorden equations, there was no relationship between a dose being in range or out of range and which patient pharmacokinetic data is used (*p* = 0.65 and 0.34 for Bugge and Reetze-Bonorden, resp.). For the Kroh equation, there was a relationship between a dose being in or out of range and which patient population data was used (*p* = 0.018); doses using normal volunteer-derived data were more likely to be within ±30% of the Aronoff doses.

## 4. Discussion

This is the first study to provide direct comparisons of the proposed dosing methodologies for a standardized patient with AKI undergoing CRRT. This study showed that calculated doses differed by 30% or more from the doses recommended by Aronoff et al., a popular dosing guideline, for over half of the antimicrobials examined. The equations frequently recommended lower doses than the dose suggested by Aronoff et al. [10]. Overall, the disparities in doses with different methods were demonstrated to be large and if applied clinically could greatly impact a patient's treatment. While the optimal dose of any of these antimicrobials is unknown, the fact that the equation-derived doses are so different from each other and from the Aronoff doses suggests that all of these sources are likely “recommending” subtherapeutic and supratherapeutic doses in many instances (Table 2).

For any of the equations, what patient populations are used as the source of the pharmacokinetic data elements made a large difference on calculated doses. For the Bugge equation, D_N_ compared to D_CKD5_ or D_AKI_ resulted in more doses within ±30% of that of Aronoff et al. (52% of D_N_ in range, 45% of D_CKD5_ in range, and 41% of D_AKI_ in range). The same result was seen with the Kroh equation (55% of D_N_ in range, 39% of D_CKD5_ in range, and 18% of D_AKI_ in range). Reetze-Bonorden found the best correlation with Aronoff et al. when utilizing AKI data (37% of D_CKD5_ in range and 50% of D_AKI_ in range). The clinical implications of these findings are intriguing. It was unexpected that using pharmacokinetic data from normal subjects would result in more doses that were within 30% of Aronoff et al. doses compared to using AKI data, yet that was our finding with the Bugge and Kroh equations. Given that neither D_AKI_ nor D_N_ led to particularly impressive agreement with Aronoff et al. doses, it is likely that the bottom line is not that use of pharmacokinetic data from patients with normal renal function is preferable to using AKI data. Rather, none of these equations performed particularly well, and perhaps therefore their use should be abandoned in favor of other individualized approaches like therapeutic drug monitoring.

Infection is the leading cause of death in AKI despite the fact that the majority of critically ill patients who receive CRRT also receive antibiotics [7]. Early, adequate antibiotic therapy has been demonstrated to be paramount to optimizing chances of survival and reducing the spread of microbial resistance [17, 18]. A recent study demonstrated the current CRRT dosing recommendations for pipercillin/tazobactam, cefepime, and ceftazidime are not sufficient to achieve pharmacokinetic and pharmacodynamic targets. Specifically, two grams twice daily of intravenous cefepime given during CRRT with ultrafiltrate rates of 2 L/h did not attain the pharmacodynamic goal (serum concentrations four times the minimum inhibitor concentration for 60–70% of the dosing interval) [19]. The cefepime dose recommendations calculated in our analysis were even lower, ranging from 1 to 3 grams daily. The Aronoff et al. text recommends one to two grams every 12 hours, which also would not meet the treatment goals. Based on the present analysis, it is probable that the current published equation-based recommendations result in underdosing of not only cefepime but many other antibiotics, as many of the calculated doses were considerably below Aronoff et al. recommendations [10].

This study has several limitations. Scarcity of pharmacokinetic data in the literature was one inherent limitation to the study and can be attributed to both the inclusion of recently approved medications which had limited available data and poor reporting of pharmacokinetic data in published studies [20]. A limitation of using any of these calculations is that they utilize reported pharmacokinetic data from a very limited number of subjects (especially when the AKI or CKD5 data are used). Making broad dosing recommendations from relatively sparse data is a potential source of error. Additionally, assumptions were made about patient size and specific CRRT flow rates and methodologies. For the purposes of calculations, the effluent rate was held constant based on the standard rates used in the Aronoff et al. text, and consequently the results reported here are only reflective of doses required with that specific CRRT effluent rate. The results may differ considerably with the use of a different effluent rate assumption and can be a future area to explore. Finally, all equation-based dosing results were compared to Aronoff et al. to aid in the comparisons between them, but there is no way to tell which dose was “the best” or would result in therapeutic concentrations.

Overall, the comparison of dosing recommendations from primarily theoretical equations to a dosing reference based on both theoretical and clinical knowledge allows insight into the existing discrepancies between different drug dosing methodologies. The disparities uncovered do not provide determination of the preferred method or the clinically correct dose but instead call to question the current utility of all CRRT drug dosing tools, particularly the methods that rely on reporting of pharmacokinetic variables in the literature. A study by Li et al. reported that an ideal dataset of parameters necessary for determining doses in CRRT are rarely included in studies, and even basic pharmacokinetic parameters such as volume of distribution and clearance are omitted in up to 20% of studies [20]. The need for diligent monitoring of medications used in CRRT patients as well as the use of sound clinical judgment when dosing is reinforced by the results of the study, and the importance of further research in the special population of individuals with AKI on CRRT is also evident. Ideally, the best solution to adjusting drug doses will be an individualized approach based on therapeutic drug monitoring and an understanding of the patient's CRRT regimen and its impact on achieving pharmacodynamic targets. However, of the drugs listed in Table 2, only vancomycin and the aminoglycosides can be routinely measured in most countries. Strategies to reach antibiotic pharmacodynamic targets more reliably have been published [21, 22] and include strategies using higher loading doses, weight-based dosing techniques, and continuous or prolonged infusions of beta-lactam antibiotics. Monte Carlo simulation of CRRT and various antibiotic doses to determine likelihood of pharmacodynamic target attainment [23] also is a promising technique to develop effective antibiotic dosing until doses are established in clinical trials. The present study indicates that CRRT antibiotic dosing based on knowledge of patient-specific pharmacokinetics and pharmacodynamic principles is preferable [21–23] to a reliance on mathematical equations.

## Supplementary Material

A list of the references used to generate the pharmacokinetic parameters responsible for the values in Table 2.

## Figures and Tables

**Table 1:** Equations proposed for determining appropriate medication dose for a patient undergoing continuous renal replacement therapy (CRRT).

Author	Equation
Bugge [4]	*Normal:* $\;D_{CRRT}=D_{N}\times \left[\frac{{CL}_{NR}}{{CL}_{N}}+\left(1-\frac{{CL}_{NR}}{{CL}_{N}}\right)\times Q_{f}\right]$
	*AKI:* $\;D_{CRRT}=D_{N}\times \left[\frac{{CL}_{AKI}}{{CL}_{N}}+\left(1-\frac{{CL}_{AKI}}{{CL}_{N}}\right)\times Q_{f}\right]$
	*CKD5:* $\;D_{CRRT}=D_{N}\times \left[\frac{{CL}_{CKD5}}{{CL}_{N}}+\left(1-\frac{{CL}_{CKD5}}{{CL}_{N}}\right)\times Q_{f}\right]$

Kroh [5]	*Normal:* $\;D_{CRRT}=D_{N}\times \frac{{CL}_{NR}+\left(Q_{f}\times S_{c}\right)}{{CL}_{N}}$
	*AKI:* $\;D_{CRRT}=D_{N}\times \frac{{CL}_{AKI}+\left(Q_{f}\times S_{c}\right)}{{CL}_{N}}$
	*CKD5:* $D_{CRRT}=D_{N}\times \frac{{CL}_{CKD5}+\left(Q_{f}\times S_{c}\right)}{{CL}_{N}}$

Reetze-Bonorden [6]	*AKI:* $\;D_{CRRT}=\frac{D_{anuric}}{1-\left(\left(Q_{f}\times S_{c}\right)/\left(Q_{f}\times S_{c}+{CL}_{AKI}\right)\right)}$
	*CKD5:* $\;D_{CRRT}=\frac{D_{anuric}}{1-\left(\left(Q_{f}\times S_{c}\right)/\left(Q_{f}\times S_{c}+{CL}_{CKD5}\right)\right)}$

D_CRRT_, dose in CRRT.

D_N_, dose in normal renal function.

D_anuric_, dose in anuric patient.

CL_NR_, nonrenal clearance in normal healthy patient.

CL_AKI_, nonrenal clearance in anuric patient with AKI.

CL_CKD5_, nonrenal clearance in anuric patient with CKD5.

CL_N_, total body clearance in normal healthy patient.

*Q*_*f*_, ultrafiltration rate.

*S*_*c*_, sieving coefficient.

**Table 2:** Antimicrobial dose recommendations for a theoretical 70 kg patient receiving CRRT at an effluent rate of 33.3 mL/min. Doses recommended in Aronoff et al. guidelines and calculated with the Bugge [4], Kroh [5], and Reetze-Bonorden [6] equations utilizing CKD5, AKI, or normal patient data are shown below. Normal text indicates a dose ≥30% less than the Aronoff dose, bold text indicates a dose ±30% of the Aronoff dose, and italicized text indicates a dose ≥30% greater than the Aronoff dose. The number of doses below, within, or above this range is reported in the final row. An asterisk (*∗*) denotes insufficient published pharmacokinetic data to allow dose calculation.

Drug name	Aronoff (mg/day)	Bugge (mg/day)			Kroh (mg/day)			Reetze-Bonorden (mg/day)	
		CKD5	AKI	Normal	CKD5	AKI	Normal	CKD5	AKI
Acyclovir	525	314	319	**399**	121	129	249	*686*	**656**
Amikacin	525	365	*∗*	**392**	348	*∗*	**389**	*1,605*	*∗*
Amoxicillin	500	*681*	*∗*	*730*	**454**	*∗*	**527**	*833*	*∗*
Amphotericin B	350	138	**269**	**317**	36	232	**304**	**390**	**356**
Ampicillin	8,000	3,348	*∗*	3,790	1,832	*∗*	2,495	3,574	*∗*
Aztreonam	2,000	*3,213*	*∗*	*3,458*	*3,183*	*∗*	*3,550*	*3,495*	*∗*
Cefazolin	4,000	1,152	*∗*	1,077	613	*∗*	500	1,333	*∗*
Cefepime	4,000	2,295	2,765	2,332	1,778	2,483	1,833	1,998	1,080
Cefotaxime	2,000	**2,399**	*∗*	*2,977*	**1,501**	*∗*	**2,367**	**2,046**	*∗*
Cefotetan	2,000	**1,533**	*∗*	1,279	1,200	*∗*	820	**1,500**	*∗*
Cefoxitin	6,000	1,739	*∗*	2,358	362	*∗*	1,290	3,258	*∗*
Ceftaroline	800	*∗*	*∗*	**605**	*∗*	*∗*	508	*∗*	*∗*
Ceftazidime	4,000	1,490	1,991	1,680	1,176	1,927	1,461	1,243	488
Ceftriaxone	2,000	826	1,102	1,131	635	1,049	1,093	**1,947**	**1,742**
Cefuroxime	2,000	**1,696**	**1,645**	**1,827**	1,377	1,300	**1,573**	*3,481*	*4,439*
Ciprofloxacin	400	*608*	*∗*	*697*	**358**	*∗*	**492**	**459**	*∗*
Clindamycin	1,800	*∗*	*∗*	**2,002**	*∗*	*∗*	**1,932**	*∗*	*∗*
Daptomycin	280	**222**	**261**	**234**	**318**	*376*	**335**	*539*	*434*
Doripenem	750	**613**	**675**	**835**	302	395	**635**	**889**	**751**
Ertapenem	1,000	*∗*	762	691	*∗*	**877**	**770**	*∗*	681
Fluconazole	400	*∗*	244	213	*∗*	*797*	*750*	*∗*	**480**
Foscavir	2,100	**2,595**	*∗*	**2,437**	**1,950**	*∗*	**1,713**	*∗*	*∗*
Ganciclovir	88	**103**	**108**	*136*	39	47	**88**	*171*	**71**
Gentamicin	120	**105**	**113**	**105**	**126**	**138**	**126**	*196*	**141**
Imipenem	2,000	1,175	1,370	**1,548**	685	977	1,245	881	718
Levofloxacin	250	*367*	*412*	*437*	**276**	*343*	*381*	*393*	*354*
Linezolid	1,200	**919**	817	**855**	**1,006**	**853**	**910**	**1,549**	*1,634*
Meropenem	3,000	1,198	1,645	1,483	642	1,312	1,069	**3,222**	2,031
Moxifloxacin	400	**336**	**476**	**342**	**358**	*568*	**368**	**472**	**443**
Nafcillin	6,000	*∗*	*∗*	**4,799**	*∗*	*∗*	**4,232**	*∗*	*∗*
Oseltamivir	150	*∗*	60	50	*∗*	23	9	*∗*	47
Piperacillin	12,000	8,112	7,891	**8,743**	6,211	5,879	7,156	**11,902**	**12,240**
Rifampin	600	*∗*	*∗*	**479**	*∗*	*∗*	**438**	*∗*	*∗*
Sulbactam	1,000	*1,855*	*∗*	*1,887*	**1,142**	*∗*	**1,190**	*1,456*	*∗*
Tazobactam	1,500	*∗*	894	933	*∗*	641	699	*∗*	**1,877**
Telavancin	500	**495**	*∗*	**383**	*840*	*∗*	*672*	*750*	*∗*
Tigecycline	100	**87**	*∗*	**89**	**82**	*∗*	**86**	**102**	*∗*
Tobramycin	120	**99**	**127**	**90**	**142**	*185*	**129**	*368*	**115**
Trimethoprim	700	*∗*	*∗*	**602**	*∗*	*∗*	**574**	*∗*	*∗*
Vancomycin	1,000	**786**	**1,184**	**896**	623	**1,220**	**788**	434	196

Below/**within**/*above* ±30% range		12/**14**/*5*	12/**9**/*1*	12/**21**/*7*	17/**12**/*2*	13/**4**/*5*	14/**22**/*4*	7/**11**/*12*	7/**11**/*4*
