# Supplementary material for: Antimicrobial Doses in Continuous Renal Replacement Therapy: A Comparison of Dosing Strategies
Source: Crit Care Res Pract. 2016 Jun 28;2016:3235765. doi: 10.1155/2016/3235765 (PMC4940534; doi:10.1155/2016/3235765)
Supplement: Supplementary file 1 — A list of the references used to generate the pharmacokinetic parameters responsible for the values in Table 2. [file 3235765.f1.docx]

| **Appendix.** Complete dataset of all medications. Shown are the total 75 medications with enough data to be used by at least one group member; seventy-one of the medications were able to be used for the Modified Kroh analysis in this study. Parameters include: D_N_ (dose in person with normal renal function based on package insert), CL_ESRD_ (total body clearance in anuric person with ESRD), CL_N_ (total body clearance in person with normal renal function), Aronoff et al. dose (dose given in Aronoff text), CL_AKI_ (total body clearance in anuric person with AKI), S_C_ (sieving coefficient), D_anuria_ (recommended dose in anuric patients based on package insert), and CL_NR_ (nonrenal drug clearance in an individual with normal renal function). Not displayed are the assumed constants: CL_CRtot_ (33ml/min; sum of renal and extracorporeal creatinine clearance in an anuric patient undergoing CRRT), CL_CRn_ (100ml/min; normal creatinine clearance for patient with normal renal function), and Q_F_ (33.3ml/min; ultrafiltration rate). Asterisk (*) denotes data was not found. | | | | | | | | | |
| --- | --- | --- | --- | --- | --- | --- | --- | --- | --- |
| **Drug Name** | **D_N_ (mg/day)** | **CL_ESRD_ (mL/min)** | **CL_N_ (mL/min)** | **Aronoff et al. Dose (mg/day)** | **CL_AKI_ (mL/min)** | **S_C_** | **D_anuria_ (mg/day)** | **CL_NR_ (mL/min)** | **References** |
| acyclovir | 800 | 28.00 | 317.00 | 525 | 31.27 | 0.60 | 400 | 78.86 | (19-27) |
| amikacin | 1050 | 2.17 | 100.00 | 525 | * | 0.93 | 350 | 6.00 | (30-33) |
| amoxicillin | 1500 | 40.00 | 220.25 | 500 | * | 0.80 | 500 | 50.75 | (37-40) |
| amphotericin B | 350 | 13.00 | 141.00 | 350 | 92.1 | 0.05 | 350 | 121.10 | (41-45) |
| ampicillin | 8000 | 30.50 | 237.94 | 8000 | * | 0.72 | 2000 | 50.20 | (39,40,46-48) |
| aztreonam | 6000 | 31.21 | 102.81 | 2000 | * | 0.70 | 2000 | 37.50 | (27,52-57) |
| cefazolin | 3000 | 5.10 | 66.60 | 4000 | * | 0.26 | 500 | 2.60 | (65-67) |
| cefepime | 6000 | 8.90 | 120.00 | 4000 | 23 | 0.80 | 500 | 10.00 | (68-70) |
| cefotaxime | 5000 | 61.80 | 280.90 | 2000 | * | 0.68 | 1500 | 110.50 | (71-73) |
| cefotetan | 3000 | 8.00 | 30.00 | 2000 | * | 0.12 | 1000 | 4.20 | (74-76) |
| cefoxitin | 5000 | 6.20 | 279.00 | 6000 | * | 0.42 | 1000 | 58.00 | (77-79) |
| ceftaroline fosamil | 1200 | * | 160.00 | 800 | * | 0.80 | 400 | 41.10 | (80-82) |
| ceftazidime | 4000 | 6.80 | 115.00 | 4000 | 28.4 | 0.81 | 250 | 15.00 | (83-85) |
| ceftriaxone | 1500 | 12.30 | 37.70 | 2000 | 22.7 | 0.11 | 1500 | 23.80 | (86-88) |
| cefuroxime sodium | 4500 | 8.24 | 125.00 | 2000 | 6.1 | 0.90 | 750 | 13.70 | (89-91) |
| ciprofloxacin | 1200 | 151.67 | 583.33 | 400 | * | 0.67 | 400 | 216.67 | (92-96) |
| clindamycin | 2250 | * | 315.00 | 1800 | * | 0.23 | 2250 | 263.03 | (97-99) |
| daptomycin | 500 | 3.40 | 11.54 | 280 | 5 | 0.16 | 210 | 3.87 | (108-113) |
| doripenem | 1500 | 28.70 | 253.89 | 750 | 44.5 | 0.67 | 500 | 85.17 | (118-123) |
| ertapenem | 1000 | * | 30.00 | 1000 | 19.3 | 0.21 | 500 | 16.10 | (128-131) |
| fluconazole | 400 | * | 18.80 | 400 | 7.8 | 0.89 | 100 | 5.60 | (138-140) |
| foscavir | 6300 | 17.50 | 148.05 | 2100 | * | 0.85 | * | 11.93 | (141-147) |
| ganciclovir | 300 | 3.50 | 246.40 | 87.5 | 10.1 | 0.85 | 18.75 | 44.12 | (148-152) |
| gentamicin | 270 | 6.03 | 72.00 | 120 | 9.16 | 0.83 | 30 | 6.00 | (153-158) |
| imipenem | 2750 | 52.50 | 371.45 | 2000 | 91.95 | 1.20 | 500 | 128.10 | (162-167) |
| levofloxacin | 750 | 37.00 | 158.00 | 250 | 51.16 | 0.64 | 250 | 59.00 | (172-178) |
| linezolid | 1200 | 76.60 | 118.00 | 1200 | 61.6 | 0.67 | 1200 | 67.17 | (184-189) |
| meropenem | 3000 | 25.40 | 255.00 | 3000 | 82.387 | 0.88 | 1500 | 61.70 | (193-198) |
| moxifloxacin | 400 | 151.67 | 200.00 | 400 | 256.67 | 0.82 | 400 | 156.60 | (172,217-220) |
| nafcillin | 6000 | * | 628.00 | 6000 | * | 0.10 | 9000 | 439.60 | (221,222) |
| oseltamivir | 150 | * | 341.10 | 150 | 33.3 | 0.58 | 30 | 0.00 | (223-228) |
| piperacillin | 16000 | 47.83 | 183.33 | 12000 | 44.025 | 0.70 | 8000 | 58.67 | (240-244) |
| rifampin | 600 | * | 198.00 | 600 | * | 0.20 | 600 | 138.00 | (256) |
| sulbactam | 4000 | 45.30 | 231.08 | 1000 | * | 0.62 | 1000 | 48.11 | (46-48) |
| tazobactam | 2000 | * | 155.90 | 1500 | 26.6 | 0.70 | 1000 | 31.18 | (240,242,244,265,266) |
| telavancin | 700 | 8.17 | 14.58 | 500 | * | 0.28 | 350 | 4.67 | (267-272) |
| tigecycline | 100 | 275.34 | 344.17 | 100 | * | 0.20 | 100 | 288.08 | (273-276) |
| tobramycin | 270 | 3.50 | 70.00 | 120 | 14.5 | 1.00 | 122.5 | 0.00 | (155,277-279) |
| trimethoprim | 1050 | * | 100.00 | 700 | * | 0.56 | 350 | 36.00 | (282) |
| vancomycin | 2000 | 6.07 | 67.67 | 1000 | 26.3 | 0.45 | 125 | 11.67 | (283-287) |
